# Integrating Mental and Physical Health Care for Low-Income Americans: Assessing a Federal Program’s Initial Impact on Access and Cost

**DOI:** 10.3390/healthcare5030032

**Published:** 2017-07-12

**Authors:** Evan V. Goldstein

**Affiliations:** Division of Health Services Management & Policy, College of Public Health, The Ohio State University, Columbus, OH 43210, USA; goldstein.292@osu.edu

**Keywords:** mental health, behavioral health integration, Federally Qualified Health Center, community health, safety net

## Abstract

Individuals with mental health disorders often die decades earlier than the average person, and low-income individuals disproportionately experience limited access to necessary services. In 2014, the U.S. Health Resources & Services Administration (HRSA) leveraged Affordable Care Act funds to address these challenges through behavioral health integration. The objective of this study is to assess the US$55 million program’s first-year impact on access and cost. This analysis uses multivariable difference-in-difference regression models to estimate changes in outcomes between the original 219 Federally Qualified Health Center (FQHC) Behavioral Health Integration grantees and two comparison groups. The primary outcome variables are annual depression screening rate, percentage of mental health and substance use patients served, and per capita cost. The results change when comparing the Behavioral Health Integration (BHI) grantees to a propensity score-matched comparison group versus comparing the grantees to the full population of health centers. After one year of implementation, the grant program appeared ineffective as measured by this study’s outcomes, though costs did not significantly rise because of the program. This study has limitations that must be discussed, including non-randomized study design, FQHC data measurement, and BHI program design consequences. Time will tell if FQHC-based behavioral–physical health care integration will improve access among low-income, medically-underserved populations.

## 1. Introduction

Integrated behavioral–physical health care remains largely under-pursued in the United States [1]. However, in July 2014, the U.S. Health Resources & Services Administration (HRSA) leveraged Affordable Care Act funding to award Mental Health Services Expansion—Behavioral Health Integration (BHI) grants to 219 Federally Qualified Health Centers (FQHCs) through a US$55 million investment. The median and mode award equaled $250,000 per grantee per year, and the purpose of the grant program was to improve and expand the delivery of behavioral health services at existing FQHCs funded under Section 330 of the Public Health Service Act. More specifically, HRSA intended to introduce and increase the coordination of mental health and substance use services at health centers located in rural and urban areas serving high-poverty and medically-vulnerable populations. While many FQHCs provided limited behavioral health services prior to the BHI grant program, HRSA acknowledged significant unmet need for behavioral health services in health centers across America [2].

HRSA structured the BHI program using Doherty, McDaniel, and Baird’s (1996) *Five Levels of Primary Care/Behavioral Healthcare Collaboration* (Five Levels) model [2]. The Five Levels model guided the BHI grantees to evaluate their current structures in light of goals for integration and set steps for change along five stages of the integration continuum: minimal, basic at a distance, basic on-site, partly integrated, and fully integrated. HRSA permitted the grantees to determine their initial level of integration based on operational capacity, acknowledging some health centers would encounter greater barriers to achieving full integration (e.g., due to physical space, hiring and system capabilities). The primary care setting is an important gateway to behavioral health services, and the further along the integration continuum a primary care organization transforms—integrating mental health and substance use treatment services into existing care processes—the better equipped the primary care organization will be to screen and treat individuals with both behavioral and physical health needs.

The initial BHI grants required the 219 FQHCs to achieve full integration of behavioral health care services into their existing medical services. Key services included implementing depression (e.g., Patient Health Questionnaire-9 [PHQ-9]) and substance use (e.g., Screening, Brief Intervention, and Referral to Treatment [SBIRT]) screenings by clinical staff during routine medical visits [2]; hiring onsite full-time counseling, psychiatry, and care coordination staff; establishing shared behavioral and physical health electronic health records; and training on integration and trauma-informed care.

### Importance and Objective

Mental and physical health comorbidity is well documented, especially the association between mental health and chronic illness [3]. More than 29% of individuals diagnosed with a medical illness also have a comorbid mental health condition, while 68% of adults with a mental illness also report to have at least one general medical disorder [4]. Physical and psychological symptoms increase together [5]. 

Comorbid physical and mental health illnesses yield lower socioeconomic status, increased health costs, and decreased length and quality of life [4]. Low-income individuals disproportionately experience poor physical health and mental illness [6], which challenges the efficacy of the traditional, fragmented health care delivery system. Indeed, the evidence suggests that integrating behavioral health services into primary care settings may improve screening and detection of adverse health conditions [1], especially among low-income populations facing burdensome barriers to accessing care [7]. The purpose of this study is thus to examine the first-year impact of HRSA’s behavioral–physical health care integration program on access and cost for low-income Americans.

## 2. Methods 

This study examined changes from FY 2014 to FY 2015 on key outcome variables among the original FQHC BHI grantees (*n* = 219). Two comparisons were made. First, to assess overarching trends, the BHI grantees were compared to the entire non-BHI FQHC population. The non-BHI grantees (*n* = 1055) provided a natural comparison group for this stage of analysis. Second, in an attempt to control for observable heterogeneity among the FQHCs, using nearest neighbor propensity score matching on the average effects on the treated, a refined comparison group was constructed from a sub-set of the non-BHI grantees (*n* = 170) that most closely matched the BHI grantees according to health center and patient population composition. Overall, the propensity score-matched cohort was statistically significantly similar to the BHI grantee cohort, as shown in Table 2. The individual FQHC was the unit of analysis. 

### 2.1. Conceptual Framework

Andersen’s (2013) revised *Behavioral Model of Health Services Use* provides the conceptual framework for this study [8]. Andersen’s model illustrates how *contextual enabling characteristics*, such as government policy intervention or program funding, can facilitate or impede the use of health care services. In this study, the HRSA BHI grant program represents an increase in contextual enabling resources available to FQHC BHI grantees. Per the Andersen model, one goal of the BHI grants is to therefore increase *potential access*. Potential access is measured in part by the available enabling resources. More enabling resources such as the BHI funding increases the likelihood for improved access to primary and behavioral health services within the BHI grantee FQHCs. Andersen’s model thus guides the development of the following hypotheses and the variables operationalized to test the hypotheses.

### 2.2. Hypotheses

This study tested four hypotheses related to access and cost.

#### 2.2.1. Access

One purpose of integrating behavioral health services is to increase access to mental health screenings through the primary care setting [1]. In this study, depression screenings are used as a mental health screening measure. Thus, compared to non-BHI grantee FQHCs, BHI grantee FQHCs will be more likely to:

**Hypothesis** **1.** Experience greater increases in depression screenings from 2014 to 2015.

Moreover, integrated care provided through FQHCs should increase access to routine mental health services that are historically difficult for low-income individuals to access [6,7]. Compared to non-BHI grantee FQHCs, BHI grantee FQHCs will be more likely to:

**Hypothesis** **2.** Experience increases in the proportion of patients seen for mental health treatment.

**Hypothesis** **3.** Experience increases in the proportion of patients served for substance use treatment.

#### 2.2.2. Cost

Although integrated care could decrease FQHC cost over time by improving care coordination and chronic illness management and preempting overutilization [9,10], initial BHI investments should increase costs after just one year of implementation. Additional services and screenings used through the integrated care model should increase marginal cost per patient within the BHI grantees. Thus, compared to non-BHI grantee FQHCs, BHI grantee FQHCs will be more likely to:

**Hypothesis** **4.** Experience greater increases in per capita costs from 2014 to 2015.

### 2.3. Independent Variables and Covariates

The primary exposure variable was a term (variable named Treated*Time) for the interaction between whether the FQHC was a BHI grantee (variable named Treated) and the time duration the BHI grantee operated its new BHI program (i.e., FY 2015, post-FY 2014 implementation; variable named Time). All regression models included a uniform set of FQHC-level covariates, including FQHC patient population, patient uninsured rate, patient race and ethnicity mix, total health center grant expenditure, and geographic region. FQHC patient population was included to control for differences in patient volume among FQHCs, measured by each FQHC’s total annual patient population. Patient uninsured rate and racial and ethnic minority rate were included to control for patient-level socioeconomic and insurance coverage-related differences associated with access [11]. Health center grant expenditure was included as a proxy to control differences in FQHC staffing and resource status. HRSA covers base staff expenditures and resources through each FQHC’s block grant reimbursement. Some organizations may generally have greater resources and/or operate in more munificent environments, which could improve their ability to adopt new programmatic innovations [12]. Geographic region was included to control for U.S. Census-level geographic variations.

### 2.4. Dependent Variables

As measures of access, the primary outcome variables were:

- Annual depression screening rate, measuring the annual percentage of patients screened for depression (variable named *Depression Screen*);
- Annual percentage of mental health patients served (variable named *Total Mental Health Patients*); and
- Annual percentage of substance use patients served (variable named *Total Substance Use Health Patients*).
- Annual per capita cost was also assessed as the primary measure of cost (variable named *Per Capita Cost*).

### 2.5. Statistical Analysis

The analysis used multivariable difference-in-difference regression models to estimate changes in outcomes associated with the BHI initiative. Separate models were estimated for each outcome measure. Models included BHI program and time-specific dummy variables. Robust standard errors were clustered at the FQHC level to correct for problems potentially caused by heteroscedasticity or serial correlation. No multicollinearity was detected. 

The regression results represent the association between the BHI program and each outcome for a full year of exposure, after adjusting for change in outcome for the control group and for the covariates. Estimated models followed the general form:

Yi = β0 + β1Treated + β2Time + β3Treated*Time + ZiCovariates + μi.(1)

The models were analyzed using ordinary least squares regression. All models were conducted for both phases of comparison, including (1) comparing the BHI grantees to the full non-BHI FQHC population; and (2) comparing the BHI grantees to the sub-comparison group derived through propensity score matching. To assess the effect of the BHI program versus non-BHI FQHCs that conducted and reported the outcomes of interest, only values greater than zero were analyzed. All missing observations were dropped from the regression analyses. Analyses were conducted using Stata version 14.0 (Stata Corp., College Station, TX, USA). 

### 2.6. Data 

This study used the U.S. Health Resources & Services Administration (HRSA) Health Center Program Grantee data gleaned from the Uniform Data System (UDS). The universe of FQHC data from FY 2014 and FY 2015 were merged. To accurately account for the impact of the 2014 BHI program, the FY 2015 data were cleaned and matched to the FY 2014 data, resulting in the exclusion of 101 health centers that were either newly-designated or newly defunct in 2015. All FQHCs were designated into four U.S. Census Bureau regions based on geographic location. Annual UDS reporting is mandatory for all FQHCs. No data or measurement discrepancies were detected.

## 3. Results

Table 1 and Table 2 show summary characteristics comparing the BHI grantees to both comparison groups at the baseline year of 2014. Overall, Table 1 illustrates that the BHI grantee cohort and the remainder of the non-BHI-grantee FQHC population significantly differ across several key characteristics. This causes concern about observable heterogeneity between the BHI grantee and the all non-BHI-grantee cohort. That said, Comparison A below reports the multivariate analysis between the BHI grantees and the full population of non-BHI-grantees to illustrate population-level trends among the FQHCs. Table 2 illustrates, however, that the propensity score-matched comparison group was significantly very similar to the BHI grantee cohort and therefore helps control for observable heterogeneity. Comparison B depicts a more refined multivariate comparison between the significantly similar BHI grantees and the propensity score-match comparison cohort.

### 3.1. Multivariate Analysis

Table 3 and Table 4 show the fully-adjusted difference-in-difference analyses. The difference-in-difference Treated*Time parameters represent adjusted percentage point (or dollar) changes from 2014 through 2015 for the BHI grantee group compared to the comparison groups (i.e., minus the difference in average outcomes in the comparison groups from 2014 to 2015), holding all other variables constant. 

#### 3.1.1. Comparison A: BHI Grantees vs. All Non-BHI-Grantee FQHC Comparison Group

In comparison to the full group of non-BHI-grantee FQHCs from 2014 to 2015, the BHI grant program was associated with a 2.8 point increase in the percentage of patients screened for depression (*p* < 0.10). The BHI program was also associated with a 1.3 point increase in the percentage of mental health patients served (*p* < 0.01) and a 0.5 point decrease in the percentage of substance use patients served, though the decrease in substance use patients was highly insignificant (*p* = 0.452). Although the BHI program was associated with a $20.29 per patient increase in per capita cost, the increase was also not statistically significant (*p* = 0.24). Holding all else constant, the covariates were generally significant. As tests of model robustness, running the models excluding insignificant covariates did not change the significance of any reported Treated*Time or Treated parameters.

#### 3.1.2. Comparison B: BHI Grantees vs. Propensity Score-Matched FQHC Comparison Group

As depicted in Table 4, the results change when comparing the BHI grantees to the propensity score-matched comparison group. Compared to the propensity score-matched cohort from 2014 to 2015, the BHI program was associated with a 3.0 point increase in the percentage of patients screened for depression; however, the parameter was insignificant (*p* = 0.22). The BHI program was associated with a 0.4 point increase in the percentage of mental health patients served, although that finding was highly insignificant (*p* = 0.548). As a result of limited statistical power, the substance use model failed to reach any significance, per the F-test for overall significance (*p* = 0.15). Again, although the BHI program was associated with a $30.52 per patient increase in per capita cost, the increase remained insignificant (*p* = 0.19). Holding all else constant, the covariates generally remained significant. As tests of model sensitivity, running the models excluding insignificant covariates did not change the significance of any reported Treated*Time or Treated parameters.

The comparison with the propensity score-match cohort washed away several significant or near-significant findings from the comparison with all non-BHI-grantee comparison. Hypothesis 1 and Hypothesis 2 were supported in the full non-BHI-grant group comparison; however, all hypotheses were ultimately unsupported in the propensity score-matched comparison. The BHI program was associated with increased depression screenings and mental health patients served, but the increases were not statistically significant in the propensity score-matched comparison. Per capita costs were not significantly higher among BHI grantees.

## 4. Discussion

Federally Qualified Health Centers play an increasingly important role in the American health care system, particularly in their pursuit of providing high-quality safety-net health care services to millions of Americans, regardless of their ability to pay or insurance status. Increased funding through the Affordable Care Act widened the health center program’s impact in communities across the U.S. While delivery systems aspiring to provide whole-person health services remain largely fragmented, especially for low-income individuals experiencing difficulties accessing behavioral health care, the idea of the FQHC BHI program seems promising. 

However, this study’s multivariate results raise several concerns about the effectiveness of the program after its first year of implementation. As Table 4 illustrates, the mere passage of time was significantly associated with increases in depression screening rates and mental health patients served. Though compared to the propensity score-matched comparison group, the BHI grant program itself did not appear to be independently responsible for improving access to behavioral health services. Time will tell if FQHC-based behavioral–physical health care integration will improve access to care for low-income, medically-underserved populations. Time will also determine the cost effectiveness of this federal program: After one year of implementation, the grant program—and requisite increases in health services intensity—was not independently responsible for significant increases in health center costs.

This study has limitations that must be discussed. The most important limitation of this study concerns the use of a nonrandomized study design. The BHI grantees were not randomly assigned to the grant funding; they applied for funding through a competitive process. To depict general trends over time, this study first depicts a multivariate analysis of the initial BHI grantees with the overall population of non-BHI-grantee FQHCs. Through propensity score matching, a statistically similar comparison group was created to help eliminate observable heterogeneity. Alas, propensity score matching still does not control for differences in unmeasured factors. Although this study attempted to control for facility-level characteristics, there may have been unmeasured variables that could have biased the results. To that end, the models estimated in this study explain relatively little variance in the outcome variables (i.e., around 10% or less). It is certainly possible that the models were not correctly or fully specified; however, it is common to observe little explainable variation in observational social science research [13]. F-tests confirm that the models provided significant estimates of the ceteris paribus relationships between the explanatory and outcome variables. The difference-in-difference approach does control for secular trends.

Measurement error can also cause endogeneity issues. One must heed caution when considering that UDS data is compiled at the FQHC level, often derived from electronic health record (EHR) systems, and submitted to HRSA by human agents. These human agents do not experience the same technical training; the turnover of some of these agents from year to year. Human agents can thus cause human error in data entry and reporting. While there is no reason to suspect any systematic human error occurred to bias the estimated parameters, this concern must be discussed when considering human data reporting. On the other hand, EHR system variation could complicate and bias BHI-related data reporting. Some EHR systems can automate and simplify data reporting, e.g., inputting data as discrete variables from which automated reports based can be run, whereas other EHR systems may require FQHC staff to laboriously enter free text data and manually export reports. One cannot disregard the possibility of systematic data-related error caused by systematic differences in EHR capability across the BHI grantees.

One must also consider the possibility that the reported results were confounded by the existence of pre-BHI grant behavioral health care service delivery. Certainly, some FQHCs—whether BHI grantees or not—provided similar behavioral health services prior to 2014, whether enabled through local funding opportunities or obligated by another program, such as the Patient-Centered Medical Home (PCMH) initiative. However, such services were likely non-systematic across particular groups of FQHCs, PCMH or otherwise. It is thus unlikely the existence of pre-2014 behavioral health services biased this evaluation. HRSA launched the BHI program in response to the significant unmet need for behavioral health services in health centers across the U.S. The BHI program was HRSA’s first major attempt to encourage systematic behavioral health integration in the FQHC setting. Moreover, this study’s regression models helped isolate individual FQHC variation and treated each cross-sectional unit as a cluster of observations over time.

In general, this analysis should be considered with caution, as the results represent just one year of post-implementation findings. The BHI grants awarded only $250,000 for new staff, services, and process changes, and this study observed only one year of post-implementation behavior. Although the BHI grantees were required to develop and submit detailed BHI grant program plans to HRSA, it is possible that not all grantees were able to execute said plans on time. For instance, perhaps not all behavioral health staff were hired as planned; perhaps not all mental health and substance use services were implemented on time. Not all BHI grantees likely achieved *full integration*. That said, such implementation pitfalls were unlikely systematic, rather random in nature.

It is also possible HRSA was misguided in structuring the BHI program using the Five Levels integration model. Given the vulnerable populations that FQHCs serve, an alternative model, such as the National Council for Behavioral Health’s *Four Quadrant Clinical Integration Model*, might have systematically prepared the BHI grantees to better consider patient population complexity and risk before devising their plans to integrate behavioral health care screenings, and change processes of care. The underlying integration model could impact the results. Needless to say, further evaluation over time will be warranted.

## 5. Conclusions

The results change when comparing the BHI grantees to a propensity score-matched comparison group versus comparing the grantees to the full population of health centers. After one year of implementation, the grant program appeared mostly ineffective as measured by this study’s outcomes, though FQHC-level per capita costs did not yet significantly rise because of the BHI program. Considering the limitations addressed above, time will tell if FQHC-based behavioral–physical health care integration will ultimately improve access among low-income, medically-underserved populations in the U.S. More time is likely needed to allow the BHI grantees to achieve full integration. As such, further evaluation will be necessary.

## Figures and Tables

**Table 1:** Summary Statistics: Behavioral Health Integration (BHI) Grantees vs. All-Non-BHI-Grantee Federally Qualified Health Center (FQHC) Comparison Group at Baseline.

Characteristic	BHI Grantees (*n* = 218)	All Non-BHI Grantee FQHC Comparison Group (*n* = 1057)	*p*-Value
Total Patients (Annual Count)	27,104	16,116	0 *
Uninsured Rate (%)	30.14	30.78	0.651
Race/Ethnic Minority Rate (%)	63.48	52.86	0 *
Health Center Grant Expenditure ($)	3,085,955	2,161,764	0 *
Region (Mean score from 1 to 4, where 1 = Midwest, 2 = Northeast, 3 = South, and 4 = West)	2.73	2.70	0.756

Mean values presented for each characteristic. *p*-Values derived through two-sample *t*-tests on difference in means. * *p* < 0.05, denoting a statistically-significant difference between the two groups.

**Table 2:** Summary Statistics: BHI Grantees vs. Propensity Score-Matched FQHC Comparison Group at Baseline.

Characteristic	BHI Grantees (*n* = 218)	Matched Comparison Group (*n* = 170)	*p*-Value
Total Patients (Annual Count)	27,104	22,586	0.128
Uninsured Rate (%)	30.14	30.85	0.696
Race/Ethnic Minority Rate (%)	63.48	62.70	0.795
Health Center Grant Expenditure ($)	3,085,955	2,780,358	0.222
Region (Mean score from 1 to 4, where 1 = Midwest, 2 = Northeast, 3 = South, and 4 = West)	2.73	2.59	0.236

Mean values presented for each characteristic. *p*-Values derived through two-sample *t*-tests on difference in means. * *p* < 0.05, denoting a statistically-significant difference between the two groups.

**Table 3:** Multivariate Regression Analysis: BHI Grantees vs. all Non-BHI-Grantee FQHC Comparison Group.

Variables	(1)	(2)	(3)	(4)
	Depression Screen Rate	Mental Health Patients	Substance Use Patients	Total Cost Per Patient
Treated	0.0354 ^	0.0245 **	0.0246 ^	$61.79
	(0.0212)	(0.0076)	(0.01322)	(41.47)
	[0.096]	[0.001]	[0.064]	[0.137]
Time	0.099 ***	0.0039 ^	−0.0028	$40.64 ***
	(0.0087)	(0.0021)	(0.0044)	(40.638)
	[0]	[0.066]	[0.523]	[0]
Treated*Time	0.0278 ^	0.013 **	−0.0056	$20.29
	(0.0162)	(0.0041)	(0.0074)	(20.13)
	[0.094]	[0.001]	[0.452]	[0.314]
Covariates				
Total Patients	−2.81 × 10^−7^	−4.47 × 10^−7^ **	−5.77 × 10^−7^ **	−$0.05 ***
	(5.47 × 10^−7^)	(1.64 × 10^−7^)	(2.43 × 10^−7^)	(0.0012)
	[0.607]	[0.007]	[0.018]	[0]
Uninsured Rate	0.0722	0.0159	−0.0111	−$304.74 ***
	(0.0468)	(0.0219)	(0.0329)	(85.59)
	[0.123]	[0.466]	[0.737]	[0]
Race/Ethnic Minority Rate	−0.0144	−0.0014	−0.0008	$261.73 ***
	(0.0248)	(0.0108)	(0.0157)	(50.65)
	[0.562]	[0.894]	[0.96]	[0]
Health Center Grant Expenditure	1.60 × 10^−9^	−1.93 × 10^−9^	−1.38 × 10^−9^	−$0
	(4.92 × 10^−9^)	(1.64 × 10^−9^)	(1.97 × 10^−9^)	(9.13 × 10^−9^)
	[0.745]	[0.239]	[0.486]	[0.344]
Region				
Northeast	0.0173	0.0097	0.0017	$177.21 **
	(0.0237)	(0.0094)	(0.0088)	(52.21)
	[0.465]	[0.3]	[0.843]	[0.001]
South	−0.0356 ^	−0.0126	−0.0009	−$26.24
	(0.0212)	(0.00876)	(0.0097)	(33.56)
	[0.086]	[0.151]	[0.93]	[0.434]
West	−0.0334	0.0018	−0.0178 ^	$290.89 ***
	(0.0216)	(0.0083)	(0.0097)	(41.46)
	[0.122]	[0.823]	[0.069]	[0]
Midwest (Reference)	+	+	+	+
Constant	0.411 ***	0.0824 ***	0.591 ***	$736.83 ***
	(0.0228)	(0.0103)	(0.0151)	(31.14)
	[0]	[0]	[0]	[0]
Observations	2392	2039	400	2481
R-squared	0.044	0.039	0.10	0.1044

Population parameter estimates generated by ordinary least squares multiple regression. All coefficients for outcome variables 1–4 represent % point changes in decimal form (i.e., 0.01 = 1% point). Robust standard errors in parentheses. *p*-Values in brackets. *** *p* < 0.001, ** *p* < 0.01, * *p* < 0.05, ^ *p* < 0.10. Source: Secondary analysis of the U.S. Health Resources & Services Administration (HRSA) Health Center Program Grantee data gleaned from the Uniform Data System (UDS). The universe of FQHC data from FY 2014 and FY 2015 were merged.

**Table 4:** Multivariate Regression Analysis: BHI Grantees vs. Propensity Score-Matched FQHC Comparison Group.

Variables	(1)	(2)	(3)	(4)
	Depression Screen Rate	Mental Health Patients	Substance Use Patients	Total Cost Per Patient
Treated	0.0327	0.0231 *	-	$5.64
	(0.031)	(0.0092)	-	(52.04)
	[0.292]	[0.01]	-	[0.914]
Time	0.1038 ***	0.0097 ^	-	$18.63
	(0.0207)	(0.0057)	-	(17.27)
	[0]	[0.087]	-	[0.281]
Treated*Time	0.0301	0.0041	-	$30.52
	(0.0249)	(0.0068)	-	(23.48)
	[0.227]	[0.548]	-	[0.194]
Covariates				
Total Patients	0	−5.82 × 10^−7^ *	-	−$0.04 *
	(6.58 × 10^−7^)	(2.35 × 10^−7^)	-	(0.0017)
	[0.382]	[0.014]	-	[0.011]
Uninsured Rate	0.0786	−0.01	-	−$529.98 ***
	(0.078)	(0.0325)	-	(138.12)
	[0.314]	[0.759]	-	[0]
Race/Ethnic Minority Rate	−0.0313	−0.0032	-	$344.85 ***
	(0.048)	(0.0223)	-	(90.31)
	[0.515]	[0.885]	-	[0]
Health Center Grant Expenditure	−8.34 × 10^−9^	−1.31 × 10^−9^	-	−$0.00001
	(8.10 × 10^−9^)	(2.96 × 10^−9^)	-	(0.00001)
	[0.304]	[0.657]	-	[0.513]
Region				
Northeast	−0.0139	0.0134	-	$173.84 **
	(0.0421)	(0.0159)	-	(60.02)
	[0.741]	[0.4]	-	[0.004]
South	−0.0208	−0.0076	-	$27.95
	(0.0378)	(0.0139)	-	(49.71)
	[0.581]	[0.587]	-	[0.754]
West	−0.0736 ^	0.0011	-	$375.66 ***
	(0.0391)	(0.0116)	-	(60.78)
	[0.06]	[0.923]	-	[0]
Midwest (Reference)	+	+	-	+
Constant	0.443 ***	0.0848 ***	-	$750.58 ***
	(0.0479)	(0.0146)	-	(60.78)
	[0]	[0]	-	[0]
Observations	733	692	154	752
R-squared	0.062	0.094	-	0.1585

Population parameter estimates generated by ordinary least squares multiple regression. All coefficients for outcome variables 1–4 represent % point changes in decimal form (i.e., 0.01 = 1% point). Robust standard errors in parentheses. *p*-Values in brackets. *** *p* < 0.001, ** *p* < 0.01, * *p* < 0.05, ^ *p* < 0.10. Source: Secondary analysis of the U.S. Health Resources & Services Administration (HRSA) Health Center Program Grantee data gleaned from the Uniform Data System (UDS). The universe of FQHC data from FY 2014 and FY 2015 were merged.
